# Mutations in the TΨC Loop of *E. coli *tRNA^Lys,3 ^Have Varied Effects on *In Trans *Complementation of HIV-1 Replication

**DOI:** 10.1186/1743-422X-4-5

**Published:** 2007-01-11

**Authors:** Wanfeng Yu, Anna McCulley, Casey D Morrow

**Affiliations:** 1 Department of Cell Biology, University of Alabama at Birmingham, Birmingham, AL 35294-0024, USA

## Abstract

**Background:**

Human immunodeficiency virus (HIV-1) exclusively selects and utilizes tRNA^Lys,3 ^as the primer for initiation of reverse transcription. Several elements within the TΨC stem loop of tRNA^Lys,3 ^are postulated to be important for selection and use in reverse transcription. The post-transcriptional modification at nucleotide 58 could play a role during plus-strand synthesis to stop reverse transcriptase from re-copying the tRNA primer. Nucleotides 53 and 54 within the TΨC stem loop of the tRNA have been shown to be important to form the complex between tRNA and the HIV-1 viral genome during initiation of reverse transcription.

**Results:**

To further delineate the features of the TΨC stem loop of tRNA^Lys,3 ^in reverse transcription, we have developed a complementation system in which *E. coli *tRNA^Lys,3 ^is provided *in trans *to an HIV-1 genome in which the PBS is complementary to this tRNA. Successful selection and use of *E. coli *tRNA^Lys,3 ^results in the production of infectious virus. We have used this single round infectious system to ascertain the effects that different mutants in the TΨC stem loop of tRNA^Lys,3 ^have on complementation. Mutants were designed within the TΨC loop (nucleotide 58) and within the stem and loop of the TΨC loop (nucleotides 53 and 54). Analysis of the expression of *E. coli *tRNA^Lys,3 ^mutants revealed differences in the capacity for aminoacylation, which is an indication of intracellular stability of the tRNA. Alteration of nucleotide 58 from A to U (A58U), T54G and TG5453CC all resulted in tRNA^Lys,3 ^that was aminoacylated when expressed in cells, while a T54C mutation resulted in a tRNA^Lys,3 ^that was not aminoacylated. Both the A58U and T54G mutated tRNA^Lys,3 ^complemented HIV-1 replication similar to wild type *E. coli *tRNA^Lys,3^. In contrast, the TG5453CC tRNA^Lys,3 ^mutant did not complement replication.

**Conclusion:**

The results demonstrate that post-transcriptional modification of nucleotide 58 in tRNA^Lys,3 ^is not essential for HIV-1 reverse transcription. In contrast, nucleotides 53 and 54 of tRNA^Lys,3 ^are important for aminoacylation and selection and use of the tRNA^Lys,3 ^in reverse transcription.

## Background

The major steps in reverse transcription of retroviral genome have been known for some time [1]. The initiation of reverse transcription occurs at the 5' end of the viral genome at a site designated as the primer-binding site (PBS) [1]. The PBS is an 18-nucleotide region that is complementary to the 3' terminal 18-nucleotides of the tRNA primer used for initiation [1-3]. The reverse transcriptase extends the bound tRNA primer from the PBS resulting in the synthesis of minus strong stop DNA [4]. The reverse transcriptase then translocates to the 3' end of the viral RNA genome and proceeds to generate a complete minus-strand DNA copy of the viral RNA genome. The RNaseH activity of the viral encoded reverse transcriptase degrades the RNA copy of the viral RNA genome. Incomplete processing of the RNA by the RNaseH activity generates RNA primers for plus-strand DNA synthesis [4]. During plus-strand synthesis, the reverse transcriptase copies the tRNA primer that is attached to the minus-strand DNA to generate a plus-strand copy of the PBS. Complementation between the plus- and minus-strand PBS facilitates the completion of the viral genome, designated as the provirus.

The vast majority of the studies that have analyzed the mechanistic events of reverse transcription have utilized *in vitro *systems comprised of tRNA, reverse transcriptase, nuclear capsid and synthetic viral RNA/DNA templates. Previous studies have found that the tRNA^Lys,3 ^and the HIV-1 genome form a complex RNA structure for initiation of reverse transcription. As a consequence of this tRNA:RNA genome interaction, the tRNA^Lys,3 ^structure is disrupted and new intramolecular bonds are formed. One important new RNA:RNA interaction is between nucleotides 53 and 54 and the first two nucleotides of tRNA^Lys,3 ^[5,6].

While *in vitro *studies have been informative in understanding the aspects of reverse transcription, they do not completely recapitulate all of the events in replication of the viral RNA genome. Our laboratory has approached this problem by generating HIV-1 proviruses that require the addition of exogenous tRNA for infectivity. In previous studies, we utilized an HIV-1 proviral genome in which the PBS had been mutated to be complementary to yeast tRNA^Phe ^[7-10]. We found that the replication of this genome could be complemented if yeast tRNA^Phe ^was supplied *in trans. In vitro *systems with synthetic tRNA/viral templates have been used to characterize many of the features of reverse transcription [11]. An important question that has been addressed using these systems is the role of modified tRNA bases that might play a role in stopping the reverse transcriptase during the plus-strand DNA synthesis to prevent complete copying of the tRNA primer. Since the completion of the proviral genome is facilitated by complementarity between the minus- and plus-strand DNA copies of the PBS, additional sequences in the plus-strand copy of the PBS as a result of copying of the tRNA primer would compromise the completion of the proviral genome. Previous studies have suggested that the methylated adenosine residue at position 58 (A58) of the tRNA could be a stop signal for the reverse transcriptase [12-14]. Support for this result comes from studies by Renda *et al*. who found that tRNA^Lys,3 ^engineered to not be methylated at A58 residue conferred a level of resistance to cells expressing this tRNA [13,14]. Additional studies though have suggested that the methylated A58 residue is not the sole stop determinant in plus-strand DNA synthesis [12].

In a recent study, we have engineered a complementation system which utilizes an HIV-1 proviral genome in which the PBS has been altered to be complementary to the 3' terminal 18-nucleotides of *E. coli *tRNA^Lys,3 ^[15]. This tRNA maintains many of the unique transcriptional modifications found in mammalian tRNA^Lys,3^, and when expressed in mammalian cells, has been shown to be aminoacylated indicating that it is fully functional [15]. Thus, this system provides an excellent opportunity to directly address the role of the modified nucleotides and tRNA structure in HIV-1 reverse transcription. In the current study, we have found that *E. coli *tRNA^Lys,3 ^with mutations at nucleotides 58, 54 and 53 in the TΨC loop region have varied effects in the production of infectious HIV-1. The results of our studies demonstrate that features in the TΨC loop of tRNA^Lys3 ^are important for the selection and use of the tRNA as a primer for HIV-1 reverse transcription.

## Materials and methods

### Tissue culture

293T cells, JC53-BL cells, and HeLa H1 cells were maintained in Dulbecco's modified Eagle's medium (DMEM) supplemented with 10% fetal bovine serum (FBS) and 1% antibiotic (Gibco/BRL, Gaithersburg, MD). All cells were cultured in 37°C incubator supplied with 5% CO_2_.

### Plasmid construction

The LS9 plasmid containing *E. coli *tRNA^Lys,3 ^gene and LS9 plasmid containing mammalian tRNA^Lys,3 ^gene were constructed as previously described [15]. The *E. coli *tRNA^Lys,3 ^gene in LS9 is located downstream of the human U6snRNA promoter. The A58 in *E. coli *tRNA^Lys,3 ^was mutated to T using the QuickChange Side-Directed Mutagenesis (Stratagene, La Jolla, CA) with Ec A58U primers: (Ec A58U forward) 5'GGTCGTGCAGGACATGAACCTGCGAC3', (Ec A58U reverse) 5'GTCGCAGGTTCATGTCCTGCACGACC3'. T54 in *E. coli *tRNA^Lys,3 ^was substituted to G with Ec T54G primers: (Ec T54G forward) 5'CAATTGGTCGCAGGATCAAGTCCTGCACGACCC3', (Ec T54G reverse) 5'GGGTCGTGCAGGACTTGATCCTGCGACCAATTG3'. T54 in *E. coli *tRNA^Lys,3 ^was also substituted to C with Ec T54C primers: (Ec T54C forward) 5'CAATTGGTCGCAGGCTCAAGTCCTGCACGACCC3', (Ec T54C reverse) 5'GGGTCGTGCAGGACTTGAGCCTGCGACCAATTG3'. TG5453 together were substituted to CC with EcTG/CC primers: (Ec TG/CC forward) 5'GACTTTTAATCAATTGGTCGCAGCCTCAAGTCCTGCACGACCC3', (Ec TG/CC reverse) 5'GGGTCGTGCAGGACTTGAGGCTGCGACCAATTGATTAAAAGTC3'. All mutations were verified by DNA sequencing.

The PBS of the proviral HIV-1 genome (NL4-3) was substituted for a PBS complementary to the 3' terminal 18-nucleotides of *E. coli *tRNA^Lys,3 ^by mutagenesis as described in [15]. A PBS shuttle vector with the substituted PBS was digested using the restriction enzymes *Bss*HII and *Hpa*I in order to release the fragment containing the *E. coli *tRNA^Lys,3 ^PBS region (an 868-bp fragment). The isolated fragment was ligated into the pNL4-3, which was also digested using the enzymes *Bss*HII and *Hpa*I. Resulting HIV-1 proviral mutant was labeled NL4-EcoLys3. Final mutants were verified by DNA sequencing.

### DNA transfections

Co-transfections were performed according to the protocol for the Fugene 6 Transfection Reagent (Roche Molecular Biochemicals, Indianapolis, IN). Briefly, 293T cells were seeded at a concentration of 2 × 10^5 ^cells per well in 6-well plates. 500 ng of NL4-EcoLys3 and 100 ng, 250 ng, 500 ng or 1000 ng LS9 plasmids encoding *E. coli *tRNA^Lys,3 ^(wild type or mutations) and 3 μl Fugene reagents were added to 100 ul of DMEM. These mixtures were incubated at room temperature for approximately 45 minutes then added drop-wise to 6-well plates. The cells were supplied with fresh media 24 hours post transfection. Supernatants were collected approximately 48 hours post transfection, centrifuged at 3,000 g, and used in JC53-BL assay to determine luciferase activity, which has been determined to correlate to units of infectious virus that is being tested. Supernatants were also assayed for HIV-1 p24 antigen (Beckman Coulter, Miami, FL).

### Analysis of virus infectivity

Serially diluted supernatants collected from co-transfections were used to infect JC53-BL cells to determine viral infectivity. JC53-BL cells were seeded 24 hours pre-infection. Infected cells were incubated for 2 hours in 37°C incubator supplemented with 5% CO_2_. After 2 hours, DMEM with 10% FBS was added to each well and the cells ere incubated for additional 48 hours. To determined luciferase activity, cells were lysed using M-PER Mammalian Protein Extraction Reagent (Pierce, Rockford, IL) and approximated 20 μL of each lysed sample were transferred to a microplate. Reporter Lysis Buffer (Promega, Madison, WI) was added to each sample in the microplate and the light intensity was measured using a Tropix TR717 Microplate Luminometer (Applied Biosystems, Foster City, CA). Uninfected cells in wells represented background luciferase activity which was subtracted from all other samples. Relative Light Units (RLu) per mL were calculated by dividing the luciferase values by their corresponding dilutions. The total amount of virus was determined by the p24 ELISA. The amount of infectious virus was determined as RLu per nanogram p24.

### Analysis of *E. coli *tRNA^Lys,3 ^expressed in mammalian cells

*E. coli *tRNA^Lys3 ^plasmids were transfected into 293T cells, and total RNA was extracted 48 hours post transfection under acidic conditions to maintain the amino acid-tRNA bond [15]. One-half of the RNA sample was treated with high pH (pH 9) to serve as a de-aminoacylated control. The samples were separated in an acidic polyacrylimide gel as previously described [15]. Northern blot was carried out with NorthernMax-Gly kit (Ambion) using previously described conditions for isolation of total RNA [15]. The probe for *E. coli *tRNA^Lys,3 ^5'GGTCGTGCAGGATTCGAACCTGCGACCAATTGATTAAAAGTCAACTGCTCTACCAACTGAGCTAACGAC3' was phosphorylated using the ready to-go kit (Amersham) with [γ-^32^P-ATP]. Hybridization was carried out under standard conditions. The blots were exposed to X-ray film which was developed using an SRX-101A developer (Konica, Wayne, NJ).

### PCR and DNA sequence analysis of the PBS regions from integrated proviruses

High molecular weight DNA (HMW) was collected using Wizard Genomic DNA Purification Kit (Promega, Madison, WI). The region encompassing the PBS was amplified by PCR using the following primers: (forward) 5'TAGACCAGATCTGAGCCTGGGAGCTC3' and (reverse) 5'CTCCTTCTAGCCTC CGCTAGTC3'. Following PCR, the products were run on 1% agarose gel and gel extracted with QIAquick Gel Extraction Kit (Qiagen, Valencia, CA) and used for DNA sequencing.

## Results

### Construction of *E. coli *tRNA^Lys,3 ^mutants and HIV-1 proviral genomes

In a recent study, we have described a complementation system which relies on the addition of *E. coli *tRNA^Lys,3 ^*in trans *to complement an HIV-1 proviral genome in which the PBS was altered to be complementary to the 3' terminal 18-nucleotides of this tRNA [15]. *E. coli *tRNA^Lys,3 ^shares many features with mammalian tRNA^Lys,3^. In a previous study, we demonstrated that transfection of the proviral genome with a PBS to *E. coli *tRNA^Lys,3 ^into mammalian cells required complementation by *E. coli *tRNA^Lys,3^cDNA in order to produce infectious virus [15]. Titration of increasing amounts of *E. coli *tRNA^Lys,3 ^plasmid resulted in corresponding increase in levels of infectious virus.

**Figure 1:**
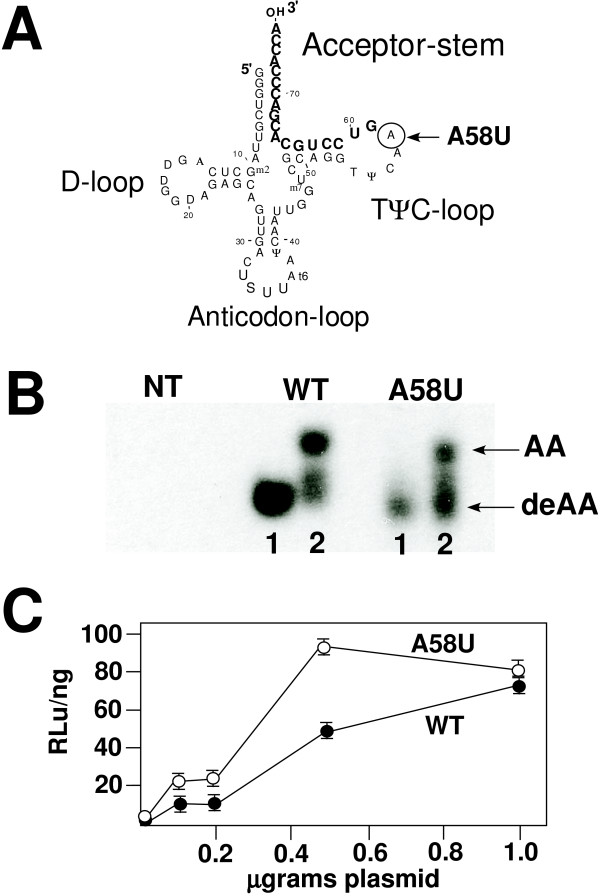
**Complementation of HIV-1 infectivity with *E. coli *tRNA^Lys,3 ^A58U mutant**. **Panel A**. Diagram of *E. coli *tRNA^Lys,3 ^A58U mutant. The base change A58U is indicated. Boldface nucleotides indicate the 3' 18-nuleotides complementary to the PBS of HIV-1 (*E. coli *tRNA^Lys,3^). **Panel B**. Expression and aminoacylation of *E. coli *tRNA^Lys,3 ^and *E. coli *tRNA^Lys,3 ^A58U in 293T cells following transfection. Total RNA was isolated under acidic conditions to stabilize the amino acid tRNA bond. Approximately one-half was treated with high pH as to break the amino acid-tRNA bond (deAA). Samples were run on an acid polyacrylamide gel and blotted into nitrocellulose. All samples were analyzed with a probe specific for tRNA^Lys,3 ^[15]. The migration of aminoacylated tRNA (Lane 2) and deacylated controls (Lane 1) are denoted as AA and deAA, respectively. NT is RNA from non-transfected 293T cells. **Panel C**. Infectivity of NL4-EcoLys3 complemented by *E. coli *tRNA^Lys,3 ^or *E. coli *tRNA^Lys,3 ^A58U. 0.5 μg NL4-EcoLys3 was co-transfected with 0.1, 0.25, 0.5, 1.0 μg plasmids encoding *E coli *tRNA^Lys,3 ^or mutant into 293T cells. Virus was collected 48 hours post transfection. The amounts of infectious virus produced from transfection were determined using the JC53-BL bioassay which measures luciferase activity [19]. The infectivity is determined by the amount of luciferase is divided by the amount of virus as determined by p24 ELISA, to give RLu per nanogram. Values are the average (+SD) from three assays.

In the current study, we have constructed mutants in the *E. coli *tRNA^Lys ^gene in which the A58 was mutated to T (A58U) (Figure 1A). To characterize the effect of this mutation we ascertained whether the tRNA could undergo aminoacylation following transfection of tRNA plasmid into mammalian cells. The capacity of the tRNA to undergo aminoacylation is indicative of proper identity elements, three-dimensional folding of the tRNA, and, most probably, inclusion into the translational machinery (Figure 1B). Following transfection of the wild type *E. coli *tRNA^Lys,3 ^and *E. coli *tRNA^Lys,3 ^A58U into mammalian cells, we analyzed these cells for the presence of aminoacylated *E. coli *tRNA^Lys,3^. The wild type and mutants were expressed in mammalian cells following transfection and aminoacylation (Figure 1B). Thus, the lack of post-transcriptional A58 modification did not influence the capacity of this tRNA to undergo expression, transport from the nucleus to the cytoplasm, aminoacylation, and presumably inclusion into the translational machinery.

Next, we wanted to determine whether the mutant *E. coli *tRNA^Lys,3 ^A58U could complement the HIV-1 proviral genome in which the PBS was altered to be complementary to the 3' terminal 18-nucleotides of this tRNA (Figure 1C). For these studies, we utilized increasing amounts of plasmids encoding the wild type and mutant *E. coli *tRNA^Lys,3 ^in the transfection. The amount of virus released from the transfections was determined using the JC53-BL assay. Complementation of the proviral genome was demonstrated following transfection of the wild type *E. coli *tRNA^Lys,3^. The levels of infectious virus increased reaching a plateau at approximately 500 ng of plasmid encoding the tRNAs. Importantly, we also found a similar level of complementation following transfection of *E. coli *tRNA^Lys,3 ^A58U, confirmed by production of infectious virus. In this case, we found no clear difference between the wild type and mutant tRNA for the capacity to complement the HIV-1 proviral genome. Analysis of the PBS from the integrated proviruses after infection revealed that all were complementary to *E. coli *tRNA^Lys,3 ^(data not shown). We found no evidence for additional tRNA sequences (after nucleotide A58) as would be expected if the reverse transcriptase had not stopped during copy of the tRNA during plus-strand synthesis. Collectively, the results of these studies show that the A58U mutation in tRNA^Lys,3 ^does not impact on the capacity of this tRNA to be selected and used in HIV-1 replication.

### Characterization of mutations within the TΨC stem loop of *E. coli *tRNA^Lys,3^

**Figure 2:**
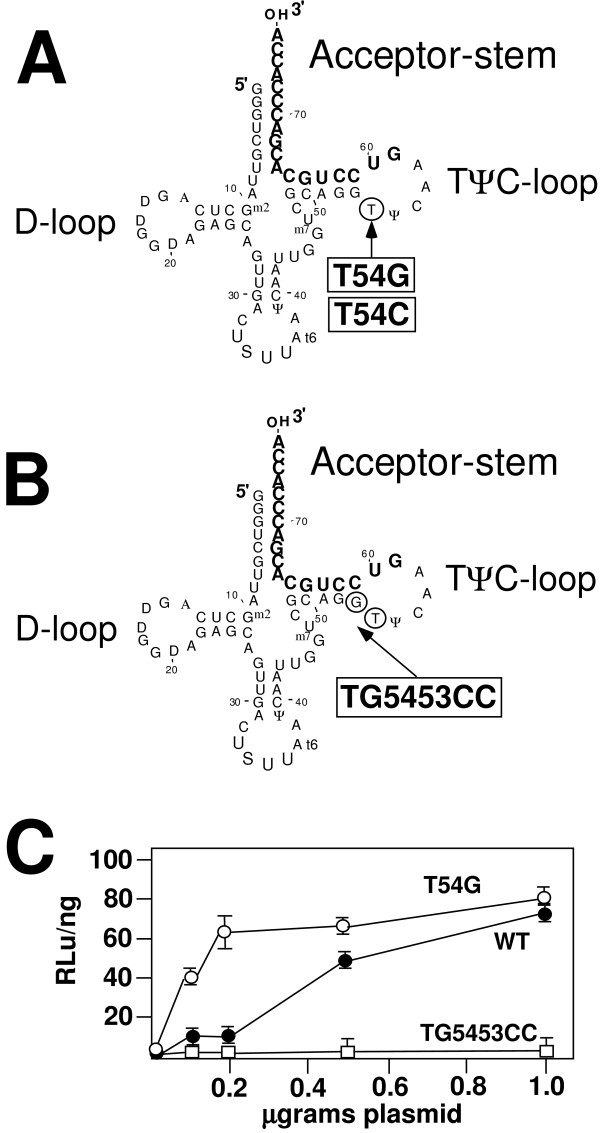
**Complementation of NL4-EcoLys3 with *E. coli *tRNA^Lys,3 ^mutants**. **Panel A**. Diagram of *E. coli *tRNA^Lys,3 ^T54G and T54C. The mutated nucleotide is indicated by a circle. **Panel B**. Nucleotide diagram *E. coli *tRNA^Lys,3 ^TG54GG. The mutated nucleotides are indicated by circles. **Panel C**. Infectivity of NL4-EcoLys3 complemented by *E. coli *tRNA^Lys,3^, *E. coli *tRNA^Lys,3 ^T54G, and *E. coli *tRNA^Lys,3 ^TG5453CC mutants. 293T cells were co-transfected with 0.5 μg of proviral plasmid and with tRNA plasmids that were titrated in at the indicated quantities. Infectivity, for complementation of plasmid NL4-EcoLys3 with *E. coli *tRNA^Lys,3 ^T54G is specified by open circle; NL4-EcoLys3 with wild type *E. coli *tRNA^Lys,3 ^is specified by closed circle; NL4-EcoLys3 with *E. coli *tRNA^Lys,3 ^TG5453CC is specified by open square. The infectivity is determined by the amount of luciferase determined by JC53-BL assay divided by amount of virus (p24 ELISA) to obtain RLu per nanogram. Values are the average (+SD) from three assays.

**Figure 3:**
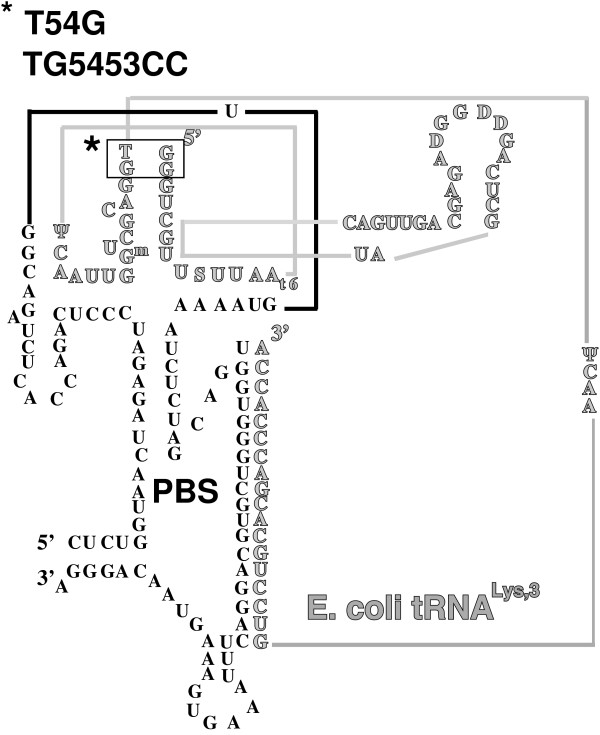
**Potential RNA secondary structure of the complex formed by NL4-EcoLys3 viral RNA and *E. coli *tRNA^Lys,3^**. The tRNA:RNA structure was adapted from that described in previous studies [5,6]. The mutations at nucleotides 54 and 53 of *E. coli *tRNA^Lys1,2 ^are boxed. The T54G mutation would be predicted to destabilize the interaction (G:G), while the TG5453CC mutation should stabilize by favorable G:C interaction.

Nucleotides within the TΨC stem loop were modeled to interact with HIV genome in forming the initiation complex between tRNA and viral RNA (Figure 3) [5,6]. A second interaction has also been found involving the tRNA and nucleotides within the stem of the TΨC stem loop that has also been termed the primer activation signal (PAS) [16]. To begin to address the importance of the TΨC stem loop, we made additional mutations in *E. coli *tRNA^Lys,3^. The first mutation targeted nucleotide 54 which is a T (Figure 2A). Two mutations were made in which the thymidine at nucleotide 54 was changed to a G or C (T54G or T54C, respectively). We also made a third mutation in which the TG at nucleotides 54 and 53 were altered to Cs (TG5453CC) (Figure 2B). Each of these mutations were postulated to affect elements of the initiation complex between the tRNA and HIV viral genome.

We first characterized the effects of these mutation on *E. coli *tRNA^Lys,3^. We determined whether the mutant tRNAs would be expressed and aminoacylated. The capacity of the tRNA to be aminoacylated correlates with the stability of this tRNA within the cell. Analysis of the aminoacylation status reveals that both T54G and TG5453CC *E. coli *tRNA^Lys,3 ^mutants were predominately aminoacylated within the cell, indicating their intracellular stability. In contrast, the T54C mutation in *E. coli *tRNA^Lys,3 ^was poorly aminoacylated and consequently expressed at a lower level than the other mutants (data not shown).

We next analyzed the capacity of these *E. coli *tRNA^Lys,3 ^mutants to complement the replication of the HIV viral genome with the PBS complementary to *E. coli *tRNA^Lys,3 ^(Figure 2C). Fixed amount of pro viral plasmid and increasing amounts of plasmids encoding wild type or mutant *E. coli *tRNA^Lys,3 ^were co-transfected into mammalian cells. The activity of the resultant virus was determined using the JC53-BL assay. The mutant T54G *E. coli *tRNA^Lys,3 ^readily complemented the infectivity of the mutant provirus at levels similar to that of the wild type tRNA. It was possible that the T54G *E. coli *tRNA^Lys,3 ^was slightly more efficient in complementation as evidenced by increased levels at lower amounts of plasmid co-transfected with the proviral genome. However, the peak levels of complementation were similar between T54G *E. coli *tRNA^Lys,3 ^and the wild type *E. coli *tRNA^Lys,3^. Not surprisingly, the mutant T54C *E. coli *tRNA^Lys,3 ^did not complement the infectivity (data not shown). Most probably this was due to poor levels of expression as a result of inability to become aminoacylated. Surprisingly, the TG5453CC *E. coli *tRNA^Lys,3 ^did not complement the mutant proviral genome, even though this tRNA was expressed and aminoacylated in cells. This lack of complementation was observed throughout the entire range of the plasmids used in the titration. Thus, the results of these studies demonstrate that mutations within the TΨC loop can impact the stability of the tRNA (T54C) as well as the capacity of this tRNA to be selected and used in HIV-1 reverse transcription. The results support a role for nucleotides 54 and 53 within the TΨC stem loop in the use of tRNA^Lys,3 ^in reverse transcription.

## Discussion

Previous studies have examined the role that post-transcriptional modification of the tRNA^Lys,3 ^have on HIV-1 reverse transcription [12-14,17]. In one study, an *in vitro *system was established which recapitulates minus-strand strong stop synthesis and the plus-strand DNA synthesis. The results from these studies establish that the modified nucleotide A58 of the natural tRNA^Lys,3 ^was only partially effective as a stop signal [12]. The reverse transcriptase in some instances could transcribe as far as the hypermodified adenosine at A37 in the anticodon loop [17]. Based on the results of these studies, the authors concluded that the modified nucleoside at A58, which is present in all tRNA^Lys,3 ^molecules, appears to be important for both the efficacy and fidelity of plus-strand DNA transfer. Renda et al., extended this work and constructed cell lines or derived MuLV based vectors, to express the A58U tRNA^Lys,3 ^[13,14]. Analysis of the replication of HIV-1 in these cells revealed that it was slower than that observed for replication of the virus in cells which did not express the mutated tRNA^Lys,3^, although the virus did eventually grow in these cells. Analysis of the resultant virus revealed that it had not undergone alteration in the PBS region. However, the inhibition of HIV-1 replication varied in individual cell clones, with some cell clones showing no inhibition. In addition, the levels of the mutated tRNA^Lys,3 ^were not determined in the individual cell lines, making it difficult to evaluate how the levels of mutant tRNA^Lys,3 ^effect viral replication and cellular metabolism. The results of these experiments suggested that mutations in A58 would have been expected to affect the capacity to produce infectious HIV-1. To further explore the potential of the A58 mutation to inhibit HIV-1 replication, we decided to determine whether the mutant tRNA would complement HIV-1 replication when provided *in trans*. For our studies, we engineered the HIV-1 proviral genome so that the PBS would be complementary to the 3' terminal nucleotides of *E. coli *tRNA^Lys,3^. In a recent study, we have shown that *E. coli *tRNA^Lys,3^, when provided *in trans *to this HIV-1 proviral genome, results in production of infectious virus [15]. If the A58 post-transcriptional modification was important for selection and use as the primer, we anticipated that co-transfection with the mutant proviral genome would not result in production of infectious virus. However, we demonstrated that the A58U mutant complemented the infectivity of the HIV-1 proviral genome at levels similar to that observed with the wild type *E. coli *tRNA^Lys,3^. At present, we cannot resolve the differences from our study with those of Renda et al [13,14]. The results of our study are consistent with the possibility that additional features of the tRNA^Lys,3 ^are more important than the modified bases for the termination of plus-strand synthesis [12]. Since our *in vivo *complementation system has all of the appropriate viral and host cell proteins available for the process of reverse transcription, which is not the case entirely for the *in vitro *system, it is possible that other protein and RNA elements can compensate for the lack of modified bases. Indeed, the *in vivo *complementation system recapitulates both the selection process as well as the events in reverse transcription. It is also likely that the three dimensional structure of the tRNA impacts on plus-strand DNA stop [12]. Previous studies have found a complex refolding of the tRNA that occurs during initiation of the minus-strand strong stop DNA synthesis [5,6]. As a consequence, new intramolecular bonds are established in the tRNA. The new tRNA structure could be a major determinant in the effective plus-strand strong stop. The viral nucleocapsid could facilitate the maintenance of the new tRNA structure. The intracellular *in trans *complementation system used in the study then recapitulates the appropriate nucleocapsid-RNA interactions, which could explain the production of infectious virus using the tRNA^Lys,3 ^mutants. Just how the modified bases affect the new three-dimensional RNA structure is unknown and will require additional studies.

To further explore the role of nucleotides in the tRNA^Lys,3 ^TΨC stem loop during reverse transcription, we made additional mutations at nucleotides 54 and 53. The T54G mutation in *E. coli *tRNA^Lys,3 ^did not affect the capacity of this tRNA to be aminoacylated or to be used in HIV reverse transcription. In contrast, mutation T54C resulted in lack of aminoacylation and consequently this *E. coli *tRNA^Lys,3 ^mutant did not complement HIV replication. Analysis of the expression level for T54C *E. coli *tRNA^Lys,3 ^revealed a decrease, upon comparison to the T54G *E. coli *tRNA^Lys,3^, and lack of aminoacylation by the lysyl-tRNA synthetase. The lower levels of expression found for tRNAs that are unable to undergo aminoacylation is consistent with previous studies that have shown unaminoacylated tRNA instability within cells [18]. An interesting result was obtained with the double mutation TG5453CC. In this case, the TG5453CC *E. coli *tRNA^Lys,3 ^was aminoacylated and generally was produced at levels similar to that of the wild type tRNA. However, this tRNA did not complement the HIV-1 proviral genome at all tRNA plasmid concentrations tested. At present, we believe that the TG5453CC mutation in tRNA^Lys,3 ^precludes or retards the tRNA from forming an initiation complex with the HIV-1 RNA genome. Previous studies have established that nucleotides with the TΨC stem loop within the *E. coli *tRNA^Lys,3 ^could be involved in two potential steps during initiation of HIV-1 reverse transcription. In the first, this region is postulated to form an intramolecular bond with the 5' end of tRNA; this RNA:RNA interaction in tRNA^Lys,3 ^is postulated to help form the structure for the initiation of HIV reverse transcription [5,6] (Figure 3). However, the T54G and TG54CC mutations could be predicted to have different effects on the intramolecular tRNA^Lys,3 ^interactions formed during initiation. The T54G mutation would be predicted to destabilize the interaction to an unfavorable base pair between nucleotide 1 and 54 (G:G). In contrast, the TG5453CC mutation was expected to promote this interaction through G:C base pairs. Thus, it was surprising that the T54G mutation in *E. coli *tRNA^Lys,3 ^still allowed complementation, while the TG5453CC mutated tRNA^Lys,3 ^did not complement. It is possible that sufficient base pair interactions still existed to form the initiation complex, but other tRNA:HIV-l genome interactions were compromised as a result of the TG5453CC mutation (Figure 3). A second function for this sequence has been found to interact with the HIV-1 genome within the U5 region (PAS) [16]. The TG5453CC mutation would have been predicted to only partially disrupt this interaction, but it could have been sufficient to inhibit initiation. Additional studies will be required to further delineate the critical intra and intermolecular interaction between the TΨC region of tRNA^Lys,3 ^and the viral genome for initiation of reverse transcription.

## Conclusion

In the current study, we have investigated the contribution that post-transcriptional modification of tRNA^Lys,3 ^at nucleotide A58 and nucleotides within the TΨC stem loop (54 and 53) have on the capacity of this tRNA^Lys,3 ^to be selected and used as the primer for HIV-1 reverse transcription. We found that these mutations, with the exception of T54C, did not affect the expression of these tRNAs and the capacity to undergo aminoacylation, and presumably, entrance into the translational processes of the cell. The capacity of these mutated tRNAs to complement HIV-1 replication, when provided intracellularly *in trans *was determined by co-transfection with a pro viral plasmid in which the PBS was mutated to be complementary to *E. coli *tRNA^Lys,3^. From the results of our studies, we conclude that the post-transcriptional modification at nucleotide 58 is not essential for function of tRNA^Lys,3 ^in HIV-1 replication. Nucleotides within the TΨC loop are also important for tRNA:HIV-l genome RNA interactions in initiation of reverse transcription. We found that one mutant, TG5453CC, could undergo aminoacylation, indicating the tRNA structure was intact, but it did not complement HIV-1 replication even though this mutant would be predicted to have favorable interactions for the initiation complex between tRNA and HIV-1 RNA. A second mutant, T54G, which would be predicted to not favor the tRNA:HIV-l genome RNA interaction in the initiation complex was fully functional for complementation. From these results, we conclude that a complex interaction between the HIV-1 viral genome in tRNA^Lys,3 ^probably involves multiple, complex RNA:RNA interactions during primer selection and reverse transcription. The absolute base pair complementarity are not necessary for these interactions, consistent with the idea the RNA structures are dynamic during HIV-1 reverse transcription.

## References

[B1] Temin HM (1981). Structure, variation and synthesis of retrovirus long terminal repeat. Cell.

[B2] Peters G, Dahlberg JE (1979). RNA-directed DNA synthesis in Moloney murine leukemia virus: Interaction between the primer tRNA and the genome RNA. J Virol.

[B3] Panet A, Berliner H (1978). Binding of tRNA to reverse transcriptase of RNA tumor viruses. J Virol.

[B4] Gilboa E, Mitra SW, Goff S, Baltimore D (1979). A detailed model of reverse transcription and tests of crucial aspects. Cell.

[B5] Isel C, Lanchy JM, Le Grice SF, Ehresmann C, Ehresmann B, Marquet R (1996). Specific initiation and switch to elongation of human immunodeficiency virus type 1 reverse transcription require the post-transcriptional modifications of primer tRNA3Lys. EMBO J.

[B6] Isel C, Marquet R, Keith G, Ehresmann C, Ehresmann B (1993). Modified nucleotides of tRNA^Lys3 ^modulate primer/template loop-loop interaction in the initiation complex of HIV-1 reverse transcription. J Biol Chem.

[B7] Kelly NJ, Morrow CD (2005). Structural elements of the tRNA Tpsi C loop critical for nucleocytoplasmic transport are important for human immunodeficiency virus type 1 primer selection. J Virol.

[B8] Kelly NJ, Morrow CD (2003). Yeast tRNA^Phe ^expressed in human cells can be selected by HIV-1 for use as a reverse transcription primer. Virology.

[B9] Yu Q, Morrow CD (2000). Essential regions of the tRNA primer required for HIV-1 infectivity. Nuc Acids Res.

[B10] Yu Q, Morrow CD (2001). Identification of critical elements in the tRNA acceptor stem and TΨC loop necessary for human immunodeficiency virus type 1 infectivity. J Virol.

[B11] Le Grice SFJ (2003). "In the beginning": Initiation of minus strand DNA synthesis in retroviruses and LTR-containing retrotransposons. Biochemistry.

[B12] Ben-Artzi H, Shemesh J, Zeelon E, Amit B, Kleiman L, Gorecki M, Panet A (1996). Molecular analysis of the second template switch during reverse transcription of the HIV RNA template. Biochemistry.

[B13] Renda MJ, Rosenblatt JD, Klimatcheva E, Demeter LM, Bambara RA, Planelles V (2001). Mutation of the methylated tRNA^Lys,3 ^residue A58 disrupts reverse transcription and inhibits replication of human immunodeficiency virus type 1. J Virol.

[B14] Renda MJ, Bradel-Tretheway B, Planelles V, Bambara RA, Dewhurst S (2004). Inhibition of HIV type 1 replication using lentiviral-mediated delivery of mutant tRNALys3A58U. AIDS Res and Human Retro.

[B15] McCulley A, Morrow CD (2006). Complementation of human immunodeficiency virus type 1 replication by intracellular selection of *Escherichia coli *formula supplied *in trans*. J Virol.

[B16] Beerens N, Groot F, Berkhout B (2001). Initiation of HIV-1 reverse transcription is regulated by a primer activation signal. J Biol Chem.

[B17] Auxilien S, Keith G, Le Grice SFJ, Darlix J-L (1999). Role of post-transcriptional modifications of primer tRNA^Lys,3 ^in the fidelity and efficacy of plus strand DNA transfer during HIV-1 reverse transcription. J Biol Chem.

[B18] Arts G-J, Kuersten S, Romby P, Ehresmann B, Mattaj IW (1998). The role of exportin-t in selective nuclear export of mature tRNAs. EMBO J.

[B19] Derdeyn CA, Decker JM, Sfakianos JN, Wu X, O'Brien WA, Ratner L, Kappes JC, Shaw GM, Hunter E (2000). Sensitivity of human immunodeficiency virus type 1 to the fusion inhibitor T-20 modulated by coreceptor specificity defined by the V3 loop of gp120. J Virol.

